# Heritability and Reversibility of DNA Methylation Induced by *in vitro* Grafting between *Brassica juncea* and *B. oleracea*

**DOI:** 10.1038/srep27233

**Published:** 2016-06-03

**Authors:** Liwen Cao, Ningning Yu, Junxing Li, Zhenyu Qi, Dan Wang, Liping Chen

**Affiliations:** 1 Department of Horticulture, College of Agriculture and Biotechnology, Zhejiang University, Hangzhou, 310058, P. R. China; 2 Zhejiang Provincial Key Laboratory of Horticultural Plant Integrative Biology, Zhejiang University, Hangzhou, 310058, P. R. China

## Abstract

Grafting between tuber mustard and red cabbage produced a chimeric shoot apical meristem (SAM) of TTC, consisting of Layers I and II from Tuber mustard and Layer III from red Cabbage. Phenotypic variations, which mainly showed in leaf shape and SAM, were observed in selfed progenies GSn (GS = grafting-selfing, n = generations) of TTC. Here the heritability of phenotypic variation and its association with DNA methylation changes in GSn were investigated. Variation in leaf shape was found to be stably inherited to GS5, but SAM variation reverted over generations. Subsequent measurement of DNA methylation in GS1 revealed 5.29–6.59% methylation changes compared with tuber mustard (TTT), and 31.58% of these changes were stably transmitted to GS5, but the remainder reverted to the original status over generations, suggesting grafting-induced DNA methylation changes could be both heritable and reversible. Sequence analysis of differentially methylated fragments (DMFs) revealed methylation mainly changed within transposons and exon regions, which further affected the expression of genes, including flowering time- and gibberellin response-related genes. Interestingly, DMFs could match differentially expressed siRNA of GS1, GS3 and GS5, indicating that grafting-induced DNA methylation could be directed by siRNA changes. These results suggest grafting-induced DNA methylation may contribute to phenotypic variations induced by grafting.

As an effective means of vegetative propagation, plant grafting is widely employed to improve tolerance to stresses or diseases, increase yield, and promote vigor. However, phenotypic variations acquired by plant grafting have been observed in a number of studies^1^,^2^,^3^,^4^,^5^,^6^. The issues of how grafting induces phenotypic variations in horticulture plants, and whether the phenotypic variations exhibit heritability remain controversial.

To date, a number of studies on whether and how grafting induces phenotypic variations have focused on the communication of DNA^7^,^8^,^9^,^10^. For example, Stegemann and Bock^9^ found that plastid DNA could be exchanged between the chimeric tissues of two tobacco plants by grafting. Recently, Fuentes *et al.*^10^ reported that entire nuclear genomes could be transferred between cells of two *Nicotiana* species via grafting. However, although cell fusion was excluded, there was no evidence supporting that the cell wall was intact during the process of callus propagation and antibiotic resistance screening in these studies^9^,^10^. In addition, no movement of DNA between cells has been detected during grafting in many experiments^11^,^12^. For example, Zhou *et al.*^11^ and Li *et al.*^12^ both failed to detect the exchange of DNA between cells via grafting. Therefore, the possibility of DNA transfer between cells with intact cell walls during grafting remains uncertain.

In contrast, the movement of endogenous small RNAs between plant cells has been demonstrated during the grafting process^12^,^13^. For example, Li *et al.*^12^ reported the transmission of small RNAs from one cell lineage to another during the grafting stage, resulting changes in the number and variety of small RNAs. Besides the communication of small RNAs during grafting, Molnar *et al.*^13^ found 24-nucleotide (24-nt) mobile small RNAs directed DNA methylation in the genome of the recipient cells via the RNA-directed DNA methylation (RdDM) pathway. Therefore, changes in DNA methylation were speculated to be induced during grafting. Additionally, this possibility gains added weight given that certain perturbations of external and internal conditions (including biotic and abiotic stresses) are known to easily induce DNA methylation modification^14^. Grafting is characterized by tight connections between cells, providing the possibility of interactions or cell communication between genetically divergent cells, resulting in the profound perturbation of the cellular environment.

DNA methylation is involved in several biological processes, including the regulation of gene expression and transposable element activity, which may induce the corresponding morphological changes without altering the DNA sequence^15^,^16^,^17^,^18^. In plants, cytosine methylation (mC) patterns have been reported to change in a spontaneous or induced manner, and are faithfully transmitted through mitosis by different DNA methyltransferase enzymes^19^. However, much less is known about whether the induced changes in DNA methylation can be passed on to the next generation. Changes in mC with high heritability were reported in some studies. For example, Johannes *et al.*^20^ demonstrated DNA methylation changes transmitted across at least eight generations without extensive DNA sequence changes in epigenetic recombinant inbred lines (epiRILs) of *Arabidopsis*. Strikingly, Cortijo *et al.*^21^ not only identified the heritability of DNA methylation changes but also pointed out that the acquired changes were responsible for heritable phenotypic alterations. Some of the differentially methylated regions (DMRs) acted as bona fide epigenetic quantitative trait loci (QTL^epi^), accounting for 60–90% of the heritability for two complex traits, flowering time and primary root length. However, Vaughn *et al.*^22^ reported that within-gene methylation was lost at a high frequency in segregating F2 families during crossing between different ecotypes in *Arabidopsis*. And it is still controversial that whether mC changes induced by the biotic and abiotic stresses are inherited to the next generation^23^. Therefore, it is essential to explore whether the induced methylation alteration can be meiotically heritable, and whether the change in DNA methylation is associated with the alteration in phenotype.

The nature of grafting is reliant on the tight connection between cells, which provides the possibility of cell communication. Chimeras are among the best materials to investigate the transmission of genetic material and the resulting phenotypic variation, especially the periclinal chimeras that possess distinct periclinal arrangements of cells with different genotypes. In our previous studies, a periclinal chimera TTC was created by *in vitro* grafting between *Brassica juncea* (tuber mustard) and *B. oleracea* (red cabbage), and phenotypic variations of the selfed progenies of TTC were observed^12^,^24^. TTC was a suitable system for analysing exchange of genetic material during grafting because the gametes were generated from the LII (T cell lineage) which was adjacent to the LIII (C cell lineage). Thus, the phenotypic variations of selfed progenies of the chimera were hypothesized to be the result of communication between different cell lineages. However, whether the cell communication can induce changes in DNA methylation, whether the induced changes in DNA methylation can be passed on to the next generation, and whether the change in DNA methylation is associated with the phenotypic variations acquired by grafting remain unknown. Therefore, exploring the changes and heritability of DNA methylation induced by grafting is the key to unravel the secret of phenotypic variations induced by grafting.

In this study, the heritability and reversibility of phenotypic variations, including leaf shape and shoot apical meristem (SAM) variation, induced by shoot apical grafting between *B. juncea* and *B. oleracea*, were observed. To unravel the mechanism of the phenotypic variation induced by grafting, the relationship between phenotypic variation and DNA methylation change was investigated. First, DNA methylation profiles of TTT and GS1 population were measured by the methylation-sensitive amplified polymorphism (MSAP) to estimate whether and to what extent grafting induced changes in DNA methylation, and several differentially methylated fragments (DMFs) were further validated by bisulfite sequencing. Second, the transmission of some DMFs from GS1 to GS5 was analysed to identify whether these acquired alterations in DNA methylation were inheritable over generations. Then, several DMFs were sequenced and their expression levels were analysed by quantitative real-Time PCR (qRT-PCR) analysis to test whether the methylation changes were associated with the phenotypic variations induced by grafting. Finally, the siRNA of TTT, GS1, GS3, and GS5 were sequenced and the differentially expressed siRNAs were blasted with the DMFs to investigate whether grafting-induced DNA methylation change was mediated by the siRNA alteration. This study will illustrate the following four questions: (1) whether and how grafting induces DNA methylation change; (2) to what extent can DNA methylation induced by grafting be transmitted between generations; (3) what is the role of induced DNA methylation alteration in the acquired phenotypic variation; (4) what is the relationship between siRNA changes and methylation changes induced by grafting? The results of this study are expected to provide a basis for understanding the phenotypic variation induced by grafting.

## Results

### Phenotypic variations in the selfed progenies of the periclinal chimera TTC

**Figure 1:**
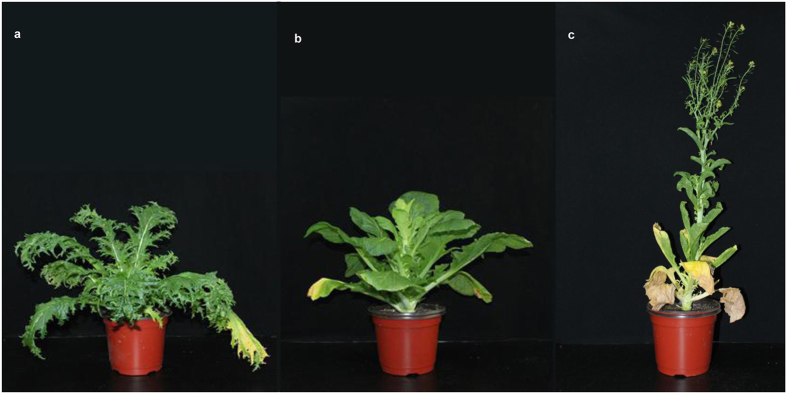
Variation in early flowering in the first selfed progeny of TTC [LI-LII-LIII, LI = outer layer of shoot apical meristem (SAM), LII = middle layer, LIII = inner layer, T = tuber mustard, C = red cabbage]. (**a**) TTT (tuber mustard); (**b**) GS1 (GS = grafting-selfing, n = generations) (blooming one week earlier); (**c**) GS1 (blooming one month earlier).

Phenotypic variations are frequently observed in grafted plants, but the issue about whether and how the variations can be passed to the next generation has not been studied well. Here, phenotypic variations were observed in the successive selfed progenies of TTC from GS1 to GS5 (see Supplementary Fig. S1). The variations were divided into two groups: leaf shape variation and SAM variation. The leaf shape variation remained consistent in the GS1 population and was propagated by self-crossing without segregation. The SAM variation showed different degrees of termination in the GS1 population and progressively decreased in the succeeding generations due to self-crossing. Further, plants with SAM variation always showed early flowering. Due to the different degrees of SAM termination, the time of early flowering also differed in GS1, from one week to one month earlier than TTT (Fig. 1). Moreover, the frequency of early flowering plants decreased gradually from GS1 to GS5. The characteristics of the leaf shape variation and SAM termination were described in detail in our previous study^12^. However, the phenotype of self-grafted plants TTT + TTT, which was produced by self grafting between tuber mustard and tuber mustard, did not show any differences when compared with TTT.

### Global DNA methylation profiles of the GS1 population

To investigate whether grafting induced changes in DNA methylation, and why the phenotype exhibited different degrees of variation within the GS1 population, DNA methylation profiles were measured by MSAP in seven individual propagated GS1 plants generated from seeds that were collected from a single TTC plant. In total, 926 clear bands were amplified from leaves using 34 pairs of selective primer combinations (see Supplementary Table S1). These amplified fragments were classified into four groups based on the presence or absence of the bands digested by specific restriction endonucleases^25^: type I (unmethylation), whose bands were present for *Eco*RI and *Hpa*II/*Msp*I combinations; type II (CHG methylation), whose bands exist only for *Eco*RI and *Hpa*II; type III (CG methylation) whose bands exist only for *Eco*RI and *Msp*I; type IV (CG/CHG methylation), representing the absence of bands for both enzyme combinations.

The total methylated ratio (types II + III + IV) and the fully methylated ratio (types III + IV) were calculated (Table 1). Compared with TTT (47.19%), the total cytosine methylation levels of the seven GS1 plants exhibited slight downward trends, and no significant changes in methylation level were observed by statistical analysis. Notably, the total methylation levels differed within GS1 individual plants, ranging from 45.03% to 46.98%. Similarly, the fully methylation levels of GS1 population also showed the same changing tendency. As expected, the methylation levels in three self-grafted plants TTT + TTT remained virtually unchanged (Supplementary Table S2).

### Changes in the DNA methylation pattern of the GS1 population

In addition to the DNA methylation levels, MSAP can also be used to investigate changes in the cytosine methylation patterns of 5′-CCGG-3′ sites. Therefore, all banding patterns between the TTT and GS1 populations were compared to obtain more detailed epigenetic differences (Table 2). All variant fragments were divided into three types: A–D represented no change; E–I represented demethylation; and J–N represented methylation. Approximately 1.62–2.81% of the amplified sites were methylated in the GS1 population when compared with those in TTT, and these percentages were lower than that of the demethylation pattern (2.92–4.21%). This result explained why the global DNA methylation levels of the GS1 population showed slight reduction. Moreover, CG hyper/hypomethylation accounted for the largest proportion of all changes in methylation pattern. In contrast to the GS1 population, only very infrequent alterations in DNA methylation pattern were observed in three self-grafted TTT + TTT plants (Supplementary Table S3).

**Figure 2:**
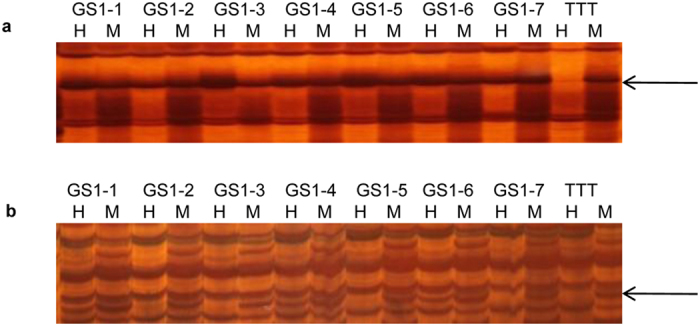
Identification of DNA methylation variation within seven GS1 individual plants by methylation-sensitive amplified polymorphism (MSAP). (**a**) uDMF (uniform DMF): the differentially methlayted sites in the GS1 population all changed in the same way compared with that in TTT. Here the fragment demethylated in all GS1 individual plants; (**b**) dDMF (distinctive DMF): the DMF at the same amplified sites exhibited distinctive methylation statuses within the GS1 population. Here the fragment demethylated only in GS1-1, GS1-4 and GS1-6, while the remaining GS1 plants exhibited the same methylation status as TTT. H indicates the band is digested by *Eco*RI and *Hpa*II, M indicates the band is digested by *Eco*RI and *Msp*I.

According to the changing characteristics of the DMFs in the GS1 population, they were divided into two groups within the seven individual GS1 plants (Fig. 2): the first group, characterized by a uniform change, was called uniform DMF (uDMF) (Fig. 2a), indicating that the differentially methylated sites in the GS1 population changed in the same way compared with those in TTT, which accounted for 42.11% (32/76). The remaining 44 DMFs at the same amplified sites exhibited distinctive methylation statuses within the GS1 population, and these DMFs were included in the second DMF group called distinctive DMF (dDMF) (Fig. 2b).

### uDMFs could exhibit long-term meiotic inheritance from generation to generation

To evaluate the meiotic heritability of DNA methylation changes induced by grafting, the transmission of all 32 uDMFs from GS2 to GS5 progenies derived from GS1 by successive self-crossing was analysed. Notably, similar to the results in the GS1 population, the uDMFs within 15 GS2, 15 GS3, 10 GS4, and 10 GS5 individual plants displayed two distinct inheritance patterns (Table 3): 24 of 32 (75%) uDMFs retained their methylation/demethylation statuses within all individual plants from GS2 to GS5, indicating that all 24 fragments were stably inherited to the GS5 generation. Nonetheless, the remaining eight uDMFs showed incomplete transmission, indicating that these fragments reverted to their original states in some plants from GS2 to GS5. Interestingly, one uDMF among the eight bands disappeared completely.

### Characterization of differentially methylated DNA sequences

To identify the molecular function of these DMFs, 25 uDMFs and 29 dDMFs out of the 76 DMFs were successfully recovered from gels and sequenced. Due to the lack of genomic information, the nucleotide sequences were blasted against BRAD (*Brassica* database) and TAIR (The *Arabidopsis* Information Resource), and all these DMFs appeared to be unigenes. It is noteworthy that the MSAP-detected genes were mainly methylated/demethylated at the transposons and exon regions of genes (Supplementary Table S4).

**Figure 3:**
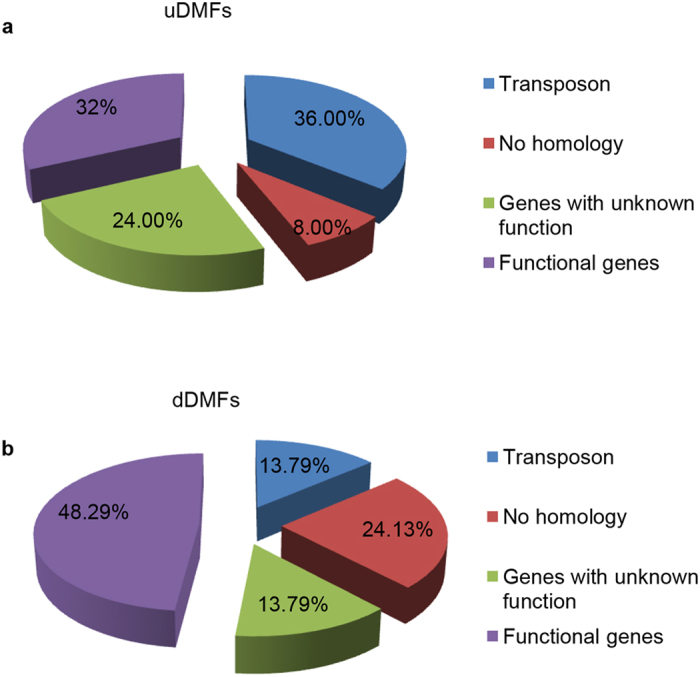
Distribution of differentially methylated genes of GS1 and the percentages of the genes in each group are listed. (**a**) A total of 25 uDMFs derived from the GS1 population were grouped into four groups; (**b**) A total of 29 dDMFs derived from the GS1 population were grouped into four groups.

Among the uDMFs, nine uDMFs (36%) were related to transposons, accounting for the largest proportion (Fig. 3a). Additionally, 32% of the uDMFs were significantly associated with homologous genes that regulated nucleotide binding, protein binding, zinc binding, stimulus response, ethylene biosynthesis, kinase activity, and transportation. Among these functional genes, one gene (uDMF32) responds to gibberellin stimulus. Interestingly, gibberellin has been demonstrated to participate in the regulation of leaf shape^26^.

Among the dDMFs (Fig. 3b), transposons only accounted for a small proportion (13.79%), while functional genes accounted for a large percentage (48.29%). These dDMFs were related to nucleotide binding, protein binding, ADP binding, threonine kinase activity, cell division, protein transport, helicase activity and metabolic process. Importantly, one dDMF (dDMF7) was homologous to phytochrome-associated serine/threonine protein phosphatase 3 (*FyPP3*), whose mutant plants display an accelerated flowering phenotype^27^. Consistent with this finding, part of the selfed progenies of TTC exhibited early flowering.

### Expression analysis of differentially methylated genes within the GS1 population

**Figure 4:**
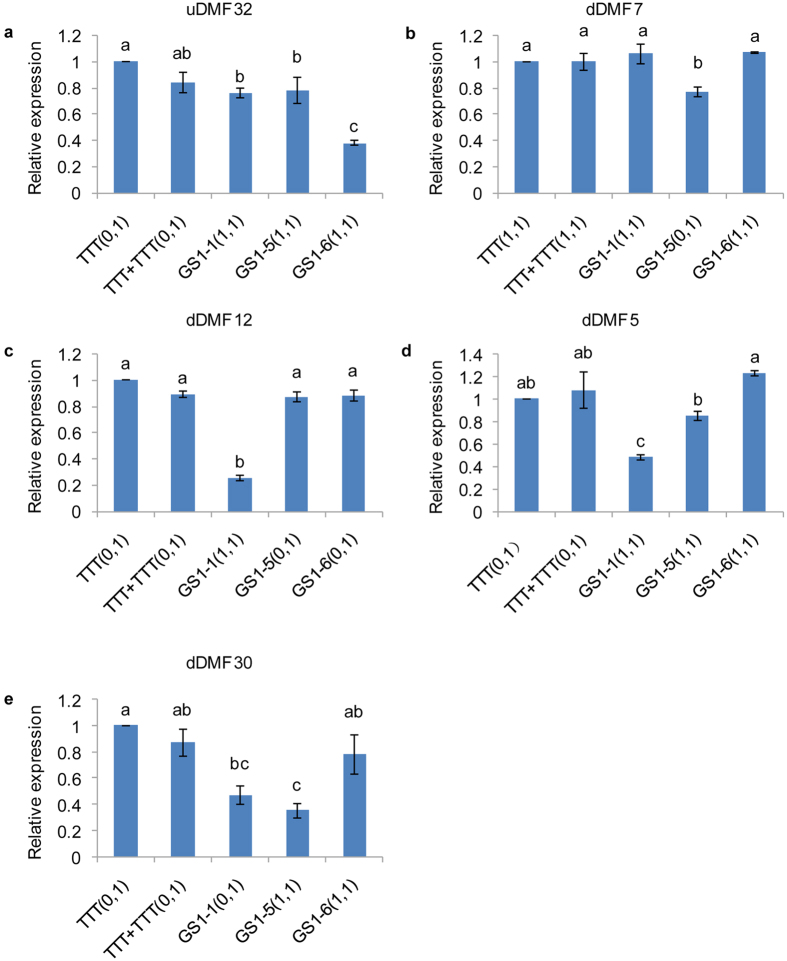
Expression analysis of five differentially methylated genes in the TTT, TTT + TTT (self-grafting between TTT and TTT), GS1-1, GS1-5, and GS1-6 plants. (**a**) uDMF32; (**b**) dDMF7; (**c**) dDMF12; (**d**) dDMF5; (**e**) dDMF30. The qRT-PCR results were analysed by the 2^−ΔΔCt^ method. Three technical replicates were included for each sample, and each bar displays the SE of triplicate assays. The values with different letters indicate significant differences at P < 0.05 using Student’s t-test.

To analyse the effect of mC variation on gene expression, qRT-PCR was used to investigate the expression levels of five genes (uDMF32, dDMF5, dDMF7, dDMF12 and dDMF30) with differentially methylated loci detected by MSAP (Fig. 4). We selected TTT, TTT + TTT and three GS1 plants, which contained all of the changing patterns for the five DMFs. As expected, uDMF32 (Fig. 4a) which was demethylated in all three GS1 plants, dDMF7 (Fig. 4b), which was *de novo* methylated in GS1-5, and dDMF12 (Fig. 4c), which was demethylated in GS1-1, all showed significant changes in expression levels between the plants with different MSAP loci respectively. However, the changes in expression levels of dDMF5 (Fig. 4d) and dDMF30 (Fig. 4e) did not show a high consistency with the changes in methylation patterns detected by MSAP. For example, dDMF5 (Fig. 4d), which had different mC patterns detected by MSAP between TTT and GS1-5, showed no significant changes in expression levels between TTT and GS1-5.

**Figure 5:**
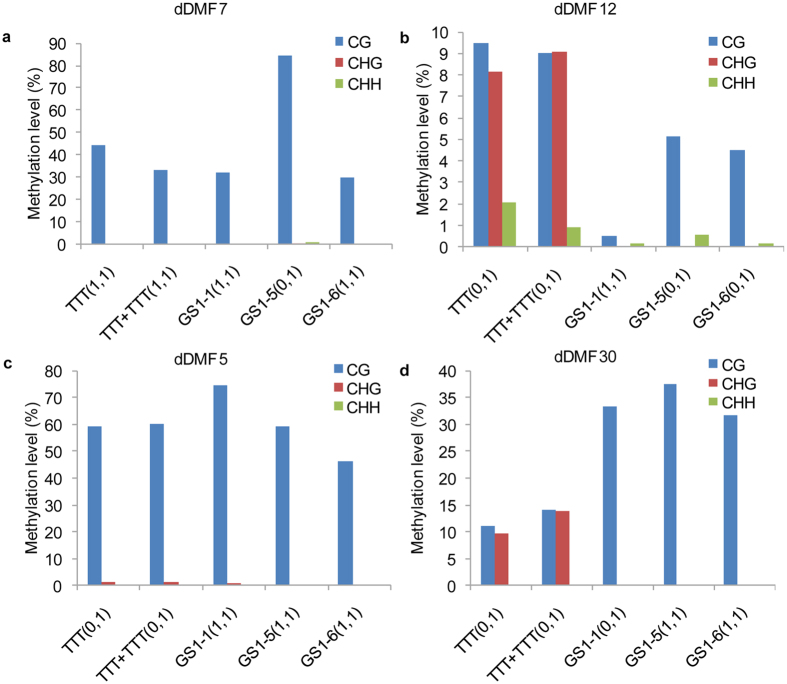
Bisufite sequencing analysis of four MSAP-detected DMFs in TTT, TTT + TTT, GS1-1, GS1-5 and GS1-6 plants. (**a**) dDMF7; (**b**) dDMF12; (**c**) dDMF5; (**d**) dDMF30.

Because the MSAP method is only used to analyse the mC variation in 5′-CCGG-3′ sites, the DMFs detected by MSAP might also contain mC variations in other methylation loci within the DMF regions. We evaluated the relationship between mC variation and gene expression at DMF regions by bisulfite sequencing analysis of the same samples (TTT, TTT + TTT, GS1-1, GS1-5 and GS1-6) as used for qRT-PCR, which could verify the MSAP-detected methylation variation as well. Here, four DMFs (dDMF5, dDMF7, dDMF12, dDMF30) were selected as representatives to perform bisulfite sequencing. Due to the lack of the genome sequences of *B. juncea*, genome walking was performed to obtain the flanking sequences of the CCGG sites of these DMFs. The bisulfite sequencing PCR results confirmed all of the DMFs identified by MSAP method. As expected, the methylation alterations of dDMF7 (Fig. 5a) and dDMF12 (Fig. 5b) regions revealed by bisulfite sequencing were in agreement with their pattern alterations detected in the MSAP method. However, although dDMF5 (Fig. 5c) was demethylated at its CCGG site in GS1-1, GS1-5, and GS1-6 as revealed by MSAP, this region displayed 15.45–28.18% CG methylation differences among the three GS1 plants. In addition, the dDMF5 region exhibited similar CG methylation levels between TTT and GS1-5, which showed different mC patterns detected by MSAP. This bisulfite sequencing result was in agreement with the transcription result of dDMF5 (Fig. 4d). Interestingly, although the bisulfite sequencing result of dDMF30 (Fig. 5d) showed CG methylation differences of 20.55% between TTT and GS1-6, the expression level of dDMF30 did not show significant changes between TTT and GS1-6.

### Mapping analysis of DMFs and differentially expressed siRNAs

**Figure 6:**
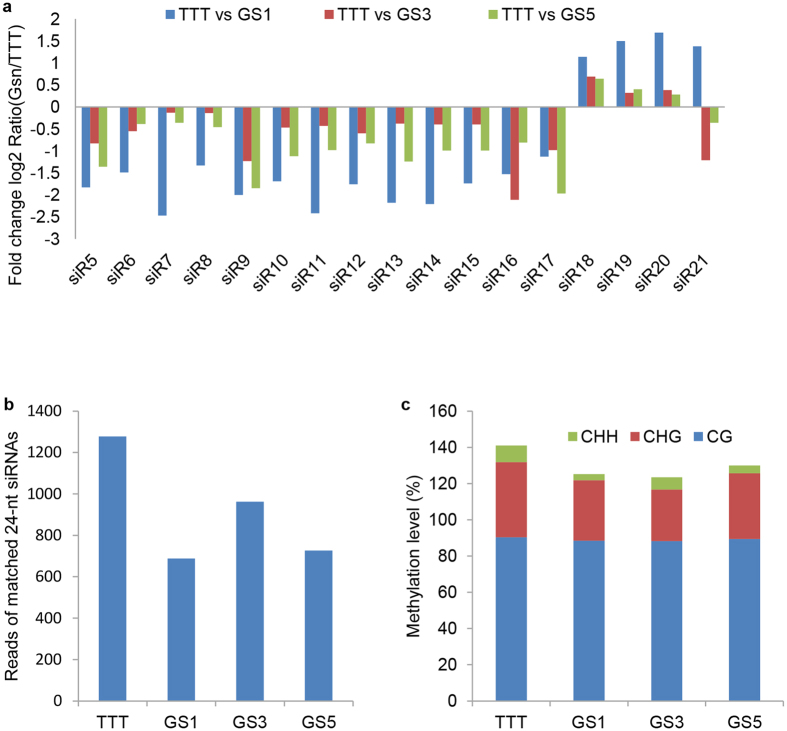
The mapping analysis of uDMF9 and differentially expressed 24-nt siRNAs between TTT and GSn (including GS1, GS3 and GS5). (**a**) the expression analysis of matched 24-nt siRNAs between TTT and GSn by small RNA sequencing; (**b**) the reads of 17 matched 24-nt siRNAs (siR5-siR21) in TTT, GS1, GS3 and GS5; (**c**) the bisulfite sequencing analysis of uDMF9 in TTT, GS1, GS3 and GS5. siRn means siRNAn, and the sequences of siRn are listed in the Supplementary Table S5.

In plant, 24-nt siRNAs usually play roles in RNA-directed DNA methylation^28^. To investigate whether grafting-induced DNA methylation changes were accompanied by siRNAs changes, eight cDNA libraries of control TTT, GS1, GS3, and GS5 were constructed for high-throughput small RNA sequencing. The small RNA sequencing data has been submitted to the NCBI Gene Expression Omnibus under accession GSE80684. A total of 11,342,348 clean reads (TTT-A), 11,592,543 clean reads (TTT-B), 11,978,407 clean reads (GS1-A), 11,336,689 clean reads (GS1-B), 11,536,016 clean reads (GS3-A), 11,584,031 clean reads (GS3-B), 11,278,937 clean reads (GS5-A), and 11,799,270 clean reads (GS5-B) were obtained by sequencing. Among the clean reads, the total reads of siRNAs were 1,038,283 (9%), 945,343 (8%), 1,089,306 (9%), 1,045,185 (9%), 1,104,833 (10%), 1,056,203 (9%), 1,022,766 (9%), and 966,597 (8%) in TTT-A, TTT-B, GS1-A, GS1-B, GS3-A, GS3-B, GS5-A, and GS5-B, respectively. Compared with the expression of siRNAs in leaves of TTT, 3284, 4789, and 6208 unique siRNAs were significantly up-regulated, and 3420, 3850, and 4245 unique siRNAs were significantly down-regulated in GS1, GS3, and GS5, respectively. Subsequently, the sequences of differentially expressed siRNAs in GS1 were mapped to all DMFs by BLAST analysis. A total of 3 DMFs (uDMF9, dDMF16, and dDMF30) were successfully matched with 82 differentially expressed siRNAs (Supplementary Table S5), and the remaining DMFs did not match to any differentially expressed siRNAs. According to the function analysis, uDMF9, dDMF16 and dDMF30 were homologous to transposable elements sequences, protein of unknown function and subtilisin-like serine endopeptidase family protein, respectively. However, the DMFs which were directly related to grafting-induced phenotypic variations did not match to any differentially expressed siRNAs between TTT and GSn. Among the matched 82 siRNAs, 17 siRNAs were 24-nt in length, and these matched 24-nt siRNAs all overlapped with the sequence of uDMF9. In addition, these 24-nt siRNAs kept the same changing pattern in expression levels in GS1, GS3 and GS5 when compared with that in TTT (Fig. 6a), and the total reads of all these matched 24-nt siRNAs were lower in GSn than those in TTT (Fig. 6b). Subsequently, to explore whether siRNAs changes affected DNA methylation in uDMF9, the bisulfite sequencing of uDMF9 was performed in TTT, GS1, GS3 and GS5. The bisulfite sequencing analysis showed that the total DNA methylation and the CHH methylation both reduced in GS1, GS3 and GS5 (Fig. 6c) when compared with that of TTT.

## Discussion

Grafting is extensively employed to improve the performance of plants in horticulture. Sometimes, unexpected phenotypic variations are observed in the progenies of grafted plants, resulting in acquired characteristics that sometimes are beneficial for agricultural production. Therefore, it is important to investigate the underlying mechanisms to make better use of the phenotypic variations induced by grafting.

Some studies have reported that the grafting-induced phenotypic variations can be inherited from generation to generation^3^,^5^,^29^. An obvious example is that the variations resulting from grafting of mung bean seedling onto the stem of a sweet potato plant were inherited steadily for 20 generations^5^. However, many studies^30^ have suggested that the phenotypic variation during grafting occurs at the physiological level and cannot be inherited in the selfed progenies of the grafted plants. Thus, whether grafting-induced phenotypic variation exhibits inheritability is controversial. In this study, we found that the variation in leaf shape remained consistent from GS1 to GS5, while SAM termination and early flowering both gradually reverted to their original states over generations. Moreover, the SAM variation displayed different degrees within GS1 individuals, although the gametes of GS1 were derived from the same T cell lineage (LII). Our results demonstrated that the heritable and reversible phenotypic variations induced by grafting could exist simultaneously in the selfed progenies of TTC.

In this study, MSAP was used to assess the extent and pattern of cytosine methylation changes induced by grafting. Because the chimera TTC consists of the genomes of both tuber mustard and red cabbage, we analysed the DNA methylation status of the selfed progeny GS1 of a single cell lineage derived from the LII of TTC. Theoretically, the DNA methylation of GS1 should be the same as that of TTT; however, our results showed genome-wide alterations in cytosine methylation levels (up to 2.16%) and patterns (up to 6.59%) in GS1 population compared with TTT, indicating that grafting could induce extensive variations in DNA methylation. What’s more, it is noteworthy that transposons accounted for a large proportion of the DMFs, which is consistent with the fact that cytosine methylation occurred more frequently in transposon sequences than in other sequences^31^,^32^,^33^,^34^.

When studying DNA methylation variation, a vital question is whether or to what extent these variations can be passed on to subsequent generations. Nowadays, the concept of transgenerational epigenetic inheritance has drawn much attention^35^,^36^,^37^. For example, in dandelions, most of the stress-induced changes in DNA methylation were faithfully transmitted to offspring^37^. Here, we found that 31.58% (24/76) of the DMFs exhibited stable meiotic inheritance for at least five generations (Table 3). Interestingly, we found that transposons accounted for the largest proportion of the stably inheritable DMFs. The result agrees with the studies reporting that the inheritance of transposon activity over multiple generations has been observed over decades in maize^38^. In addition, we found the remaining 68.42% DMFs reverted over generations. For the reversible DMFs, it is possible that the DMFs affected only one homologous chromosome of the T cells located in the layer II of TTC during the grafting stage, and were segregating in the selfed progenies, as observed in a previous study^39^. Alternatively, it is possible that some loci easily experience the cycles of forward and reverse variation. For example, DNA methylation changes constantly from one generation to the next generation in *Arabidopsis*; however, the level of changes in DNA methylation does not exhibit a linear increase over generations^40^,^41^. Hence, it has been proposed that some epimutations are not stably inherited due to recurrent cycles of forward and reverse variation. This finding that the DNA methylation changes induced by grafting can be inheritable and reversible is similar to the observation that the hereditability and reversibility of the phenotypic variations induced by grafting can exist simultaneously in the selfed progenies of TTC. However, the relationship between the DNA methylation and phenotypic variation during grafting is unclear.

Then, the molecular analysis of DMFs indicated that the DNA methylation variation induced by grafting mainly occurred within transposons and exon regions of genes. DNA methylation has been reported to participate in the regulation of transposon silencing and activation^42^,^43^. Transposons will be activated and mobilized if they undergo demethylation, and the demethylated transposed elements may affect gene expression when they are inserted upstream or downstream of the coding genes^44^. In terms of coding genes, two genes, which might be associated with grafting-induced phenotypic variations in leaf shape and early flowering, attracted our attention. The first was *FyPP3* (dDMF7), encoding the catalytic subunit of the serine/threonine protein phosphatase 2A, whose mutant plants exhibit accelerated flowering^27^. The second gene (uDMF32) was homologous to At2g39540, a gibberellin-regulated family protein that responds to gibberellin stimulus. It is well known that gibberellin influences leaf development, including leaf shape^26^. DNA methylation is known to regulate the expression levels of coding genes^17^. Here, we found the coding genes, including dDMF7 and uDMF32, with differentially methylated loci showed differential gene expression, suggesting that DNA methylation induced by grafting might play a role in the induced phenotypic variation. However, the expression analysis of all five selected DMFs revealed that the correlations between gene expression and methylation changes detected by MSAP were complicated. One reason is that apart from the differentially methylated loci within DMFs detected by MSAP, there are other variable loci located within the regions, and the CG sites within DMF regions are limited. For example, although MSAP revealed that dDMF30 (Fig. 5d) was demethylated at its CCGG site in the GS1 population, the overall level of CG methylation in this region was higher in the GS1 population than in the TTT plant. Alternatively, the role of DNA methylation depends on its location of a gene. As reported, DNA methylation located in the promoter seems to be negatively correlated with gene expression; however, the association becomes unclear when it comes to the DNA methylation of the gene-body^22^,^45^,^46^.

*In vitro* apical grafting is characterized by a tight connection between cells, providing the possibility of interactions or cell communication between different cell lineages, and resulting in the profound perturbation of the cellular environment. The chimera TTC may undergo grafting stress during grafting between tuber mustard and red cabbage. DNA methylation is known to be sensitive and responsive to stresses, including internal and external perturbations^14^, and the methylation changes are immediate and crucial for helping them adjust to stress. The chimera TTC is speculated to try to adapt to the perturbation (cell to cell communication) caused by grafting by varying its DNA methylation. Particularly, the overall DNA methylation levels of the GS1 (45.03–46.98%) population were slightly decreased compared with TTT (47.19%), which is consistent with previous studies reporting that environmental stresses tend to cause demethylation of genomic DNA^47^,^48^,^49^,^50^.

It was previously reported that small RNAs, especially the 24-nt siRNAs, from one parent plant could move across the graft union via plasmodesmata and phloem to regulate novel targets in the genome or transcriptome of the opposite parent plant during grafting, such as RdDM^51^,^52^. Our previous study^12^ reported that heterogeneous small RNAs were transported from the red cabbage cells to the tuber mustard cells in chimera TTC, and apart from transmission, the expression of small RNAs changed significantly during the grafting stage, indicating that the communication and perturbation of heterogenous cells led to the fluctuation of variety and expression of small RNAs during grafting. That means the grafting is a complicated process which is involved in cell communication and stress. Here, we found that the expression of siRNAs altered dramatically in the selfed generations of TTC, indicating the grafting-induced changes in expression levels of siRNAs were observed not only in the grafting stage but also in the selfed progenies of grafted plant. Some differentially expressed 24-nt siRNAs were successfully mapped to the sequence of DMFs, and the expression levels of mapped 24-nt siRNAs decreased in the GSn compared with TTT, which was consistent with the decreased CHH methylation levels of the matched DMF in GSn, indicating that the grafting-induced DNA methylation variation could be affected by the RdDM pathway. Furthermore, the inheritable variation in DNA methylation might be maintained by the differentially expressed siRNAs through RdDM after grafting, because the differentially expressed siRNAs retained the same changing patterns in the successive progenies of TTC. However, due to the fact that MASP technique allows identification of the DNA methylation changes only within CCGG sequence, the differentially methylated regions detected by MSAP are less than the really existing ones, which may result in the relative low number of matched siRNAs and no DMFs directly associated with phenotypic variations matching to any differentially expressed siRNAs between TTT and GSn.

## Conclusion

In conclusion, *in vitro* apical grafting between *B. juncea* and *B. oleracea* induced phenotypic variation, mainly including leaf shape and SAM variation, of the selfed progenies of the chimera TTC, which exhibited both hereditability and reversibility. To explore the mechanism of the grafting-induced phenotypic variation, the relationship between phenotypic variation and alteration in DNA methylation was investigated. Extensive changes in DNA methylation were identified by MSAP in GS1 population compared with TTT. Some of the DNA methylation changes were inherited for at least five generations, while other DNA methylation alterations gradually reverted to their original states over generations. In addition, these differentially methylated loci mainly focused on the transposons and the exon regions of genes, which further affected gene expression of phenotypic variation-related genes. Finally, the expression levels of siRNA were found to change significantly in GS1, GS3 and GS5 compared with those in TTT, and some differentially expressed siRNAs could match to the DMFs. These results suggest that DNA methylation alteration induced by grafting may play a role in the phenotypic variations observed during grafting and that grafting-induced DNA methylation variation can be directed by siRNA changes, which provide a basis for exploring the inheritance mechanism of the phenotypic variation induced by grafting.

## Materials and Methods

### Plant material

The periclinal chimera TTC [LI-LII-LIII, LI = outer layer of shoot apical meristem (SAM), LII = middle layer, LIII = inner layer, T = tuber mustard, C = red cabbage] used in this study was created by *in vitro* grafting between tuber mustard [*Brassica juncea* (L.) Czern. et Coss. var. *tumida* Tsen et Lee] and red cabbage (*B. oleracea* L. var. *capitata* L.) which were named as TTT and CCC respectively. In the experiment, the tuber mustard is an inbreed line^24^. The nuclear DNA contents of TTC were measured by flow cytometry as previously described and were indistinguishable from the mechanical mixture of TTT and CCC (see Supplementary Fig. S2)^53^. Thus, cell fusion induced by cell to cell grafting was excluded in the experimental material, and DNA transfer between the T and C cell lineages was also excluded. Starting in 2007, the first self-crossed line derived from a single TTC plant was named GS1, and the successive generations GS2, GS3, GS4 and GS5 were derived from GS1 through self-crossing. Meanwhile, an *in vitro* grafting between tuber mustard and tuber mustard, named TTT + TTT, was synchronously generated as a control. All materials used for the MSAP analysis are listed in Supplementary Fig. S3.

### Analysis of MSAP and DMFs

Genomic DNA was extracted from the fourth leaf at the four-leaf stage using the CTAB method described by Murray and Thompson^54^. Quality and quantity of extracted DNA was measured using NanoDrop 2000 (Thermo, Waltham, US). MSAP analysis was performed as previously reported^55^. The primers used for each procedure are provided in Supplementary Table S1. To avoid technique biases, we performed three technical replicates for each sample. Additionally, the upper and lower bands of the gel were not scored in the final data because these parts were always unstable. Statistical analysis of the methylation level differences between TTT and GS1 was performed as previously described^56^. The DMFs were isolated from the polyacrylamide gels and incubated at 94 °C for 10 min prior to the addition of 20 μl of TE buffer. Then, the PCR reaction was performed using the corresponding primers with 1 μl of eluted DNA. The PCR products were purified using the Agarose Gel DNA Extraction kit (Takara, Ostu, Japan). Subsequently, the purified PCR product was cloned into the pMD18-T vector (Takara) and sequenced. All DNA sequences were blasted against BRAD and TAIR.

### RNA isolation and quantitative real-Time PCR Analysis

Total RNA was extracted using Trizol kit (Invitrogen, Carlsbad, CA) from the same leaf tissues from TTT, TTT + TTT, GS1-1, GS1-5, and GS1-6 used for the DNA isolation. The NanoDrop 2000 (Thermo) and a 1.2% agarose gel were used to detect the quality and quantity of the RNA. cDNA synthesis was performed using the PrimeScript RT reagent kit (Takara). The qRT-PCR was performed using 20 μl volumes of SYBR (Takara) and the ABI STEPONE Real-Time PCR system as previously reported^12^. All gene-specific primers for qRT-PCR analysis are listed in Supplementary Table S1. The 25S ribosomal RNA gene was selected as a reference gene for normalization. Three technical replicates were included for each sample. Finally, the statistical significance of differences in expression was analysed with SPASS 19.0 software.

### Genome walking of the DMFs

The flanking regions of the DMFs were amplified from a DNA template of TTT using Genome walking Kit (Takara), according to the manufacture’s instruction. The specific primers for the four DMFs are listed in Supplementary Table S1. The PCR products were purified using the Agarose Gel DNA Extraction Kit (Takara) and then cloned into the pMD18-T vector (Takara) and sequenced.

### Bisulfite sequencing

A total of 1 μg of the DNA extracted from each sample was modified using the EZ DNA Methylation-Gold kit (Zymo Research, Orange County, US) as recommended. The PCR reaction was performed with 2 μl of treated DNA in a 25 μl reaction system under the following conditions: 5 min at 94 °C, followed by 30 cycles of 30 s at 94 °C, 30 s at 58 °C, and 30 s at 72 °C, with a final 5 min elongation at 72 °C. The primers for the differentially methylated loci were designed using the MethPrimer tool (http://www.urogene.org/cgi-bin/methprimer/methprimer.cgi) and are listed in Supplementary Table S1. The PCR products were purified using the Agarose Gel DNA Extraction kit (Takara). Subsequently, the purified PCR products were cloned into the pMD18-T vector (Takara), and a minimum of 10 clones for each sample were sequenced. The detailed DNA methylation state was analysed using the online software Kismeth (http://katahdin.mssm.edu/kismeth/revpage.pl).

### Small RNA sequencing and mapping

The fourth leaves of all samples (TTT, GS1, GS3 and GS5) were collected at the four-leaf stage (two biological replicates per sample combined 3 plants each). All samples were sent to the Beijing Genomics Institute (BGI, Beijing, China) for sequencing. A total of eight cDNA libraries were sequenced. For each sample, the 18–30 nt small RNA fragments were purified, ligated to the 5′ and 3′ adaptors, converted to cDNA by reverse transcription PCR, and subjected to PCR amplification. Small RNA libraries were sequenced using the Illumina-HiSeq. After eliminating the contaminant reads and low quality reads, tags were used to predict siRNA using tag2siRNA. Differentially expressed siRNAs were identified using the DEGseq (an R package to identify differentially expressed genes from data)^57^. The fold change was calculated via log_2_-ratio. siRNAs exhibiting a |fold change| ≥ 1 and adjusted g-value < 0.05 were selected as differentially expressed siRNAs. The differentially expressed siRNAs and DMFs were blasted via NCBI (http://blast.ncbi.nlm.nih.gov/Blast.cgi).

## Additional Information

**How to cite this article**: Cao, L. *et al.* Heritability and Reversibility of DNA Methylation Induced by *in vitro* Grafting between *Brassica juncea* and *B. oleracea. Sci. Rep.* **6**, 27233; doi: 10.1038/srep27233 (2016).

## Supplementary Material

Supplementary Information

## Figures and Tables

**Table 1:** Analysis of DNA methylation levels detected by methylation-sensitive amplified polymorphism (MSAP) in the parental plant TTT and seven individual GS1 (GS = grafting-selfing, n = generations) plants.

MSAP Band type	TTT	GS1-1	GS1-2	GS1-3	GS1-4	GS1-5	GS1-6	GS1-7
I (unmethylation)	489	503	508	509	499	497	506	491
II (CHG methylation)	140	135	134	132	134	136	132	135
III (CG methylation)	292	283	281	280	289	288	284	295
IV (CG/CHG methylation)	5	5	3	5	4	5	4	5
Total Bands	926							
Total methylated bands^a^	437	423	418	417	427	429	420	435
Total methylation ratio (%)^b^	47.19	45.68	45.15	45.03	46.11	46.33	45.36	46.98
Full methylated bands^c^	297	288	284	285	293	293	288	300
Full methylated ratio (%)^d^	32.07	31.10	30.67	30.78	31.64	31.64	31.10	32.40

^a^Total methylated bands = II + III + IV;

^b^Total methylated ratio = [(II + III + IV)/(I + II + III + IV)] × 100%;

^c^Fully methylated bands = III + IV;

^d^Fully methylated ratio = [(III + IV)/(I + II + III + IV)] × 100%.

**Table 2:**
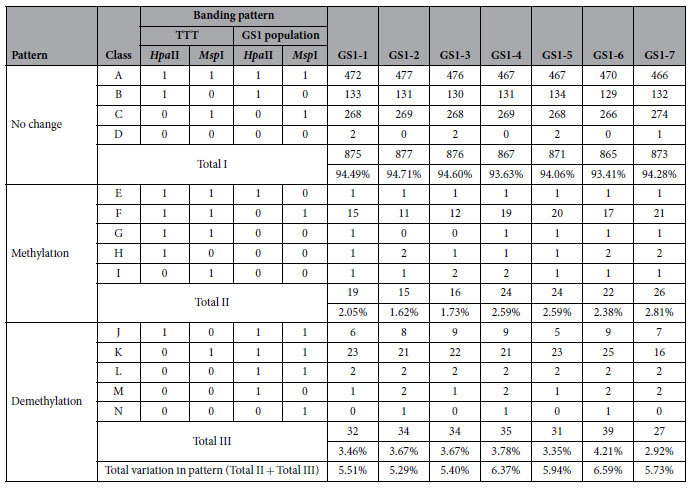
Analysis of cytosine methylation pattern variations in seven GS1 individual plants compared with control TTT.

A score of 1 and 0 represents presence and absence of bands, respectively. Values in parentheses indicate percentage of bands in each pattern which was determined by dividing number of bands in each pattern by total number of bands in all three patterns.

**Table 3:** Analysis of meiotic inheritance of 32 uDMFs from the GS1 to GS5 generations.

	Variation pattern		Inheritance ration of uDMFs (GS1 to GS5) (%)			
	TTT	GS1	GS1-GS2	GS2-GS3	GS3-GS4	GS4-GS5
uDMF1	(0, 1)	(1, 1)	86.67 (13/15)	33.33 (5/15)	10.00 (1/10)	30.00 (3/10)
uDMF2	(0, 1)	(1, 1)	60.00 (9/15)	60.00 (9/15)	30.00 (3/10)	40.00 (4/10)
uDMF3	(0, 1)	(1, 1)	60.00 (9/15)	53.33 (8/15)	30.00 (3/15)	20.00 (2/10)
uDMF4	(0, 1)	(1, 1)	20.00 (3/15)	13.33 (2/15)	50.00 (5/10)	20.00 (2/10)
uDMF5	(1, 1)	(0, 1)	93.33 (14/15)	60.00 (9/15)	60.00 (6/10)	60.00 (6/10)
uDMF6	(1, 1)	(0, 1)	93.33 (14/15)	20.00 (3/15)	70.00 (7/10)	80.00 (8/10)
uDMF7	(1, 1)	(0, 1)	60.00 (9/15)	53.33 (8/15)	20.00 (2/10)	30.00 (3/10)
uDMF8	(0, 0)	(1, 0)	0.00 (0/15)	0.00 (0/15)	0.00 (0/10)	0.00 (0/10)
uDMF9	(1, 1)	(0, 1)	100.00	100.00	100.00	100.00
uDMF10	(1, 1)	(1, 0)	100.00	100.00	100.00	100.00
uDMF11	(1, 1)	(0, 1)	100.00	100.00	100.00	100.00
uDMF12	(1, 1)	(0, 1)	100.00	100.00	100.00	100.00
uDMF13	(1, 1)	(0, 1)	100.00	100.00	100.00	100.00
uDMF14	(0, 1)	(1, 1)	100.00	100.00	100.00	100.00
uDMF15	(0, 1)	(1, 1)	100.00	100.00	100.00	100.00
uDMF16	(1, 0)	(1, 1)	100.00	100.00	100.00	100.00
uDMF17	(0, 1)	(1, 1)	100.00	100.00	100.00	100.00
uDMF18	(0, 0)	(1, 1)	100.00	100.00	100.00	100.00
uDMF19	(1, 1)	(0, 1)	100.00	100.00	100.00	100.00
uDMF20	(0, 1)	(1, 1)	100.00	100.00	100.00	100.00
uDMF21	(0, 1)	(1, 1)	100.00	100.00	100.00	100.00
uDMF22	(0, 1)	(1, 1)	100.00	100.00	100.00	100.00
uDMF23	(1, 0)	(1, 1)	100.00	100.00	100.00	100.00
uDMF24	(1, 1)	(0, 1)	100.00	100.00	100.00	100.00
uDMF25	(0, 0)	(1, 1)	100.00	100.00	100.00	100.00
uDMF26	(1, 0)	(0, 0)	100.00	100.00	100.00	100.00
uDMF27	(1, 0)	(1, 1)	100.00	100.00	100.00	100.00
uDMF28	(1, 0)	(1, 1)	100.00	100.00	100.00	100.00
uDMF29	(0, 1)	(1, 1)	100.00	100.00	100.00	100.00
uDMF30	(1, 0)	(1, 1)	100.00	100.00	100.00	100.00
uDMF31	(0, 1)	(0, 0)	100.00	100.00	100.00	100.00
uDMF32	(0, 1)	(1, 1)	100.00	100.00	100.00	100.00

uDMF: uniform DMF; DMF: differentially methylated fragment. A score of 1 and 0 represents presence and absence of bands, respectively. (1, 1): unmethylation; (1, 0): CHG methylation; (0, 1): CG methylation; (0, 0): CG/CHG methylation.
