# Supplementary material for: Heritability and Reversibility of DNA Methylation Induced by in vitro Grafting between Brassica juncea and B. oleracea
Source: Sci Rep. 2016 Jun 3;6:27233. doi: 10.1038/srep27233 (PMC4891673; doi:10.1038/srep27233)
Supplement: Supplementary Information [file srep27233-s1.pdf]

Heritability and Reversibility of DNA Methylation Induced by *in vitro* Grafting between *Brassica juncea* and *B. oleracea*

Liwen Cao[1, 2], Ningning Yu[1, 2], Junxing Li[1, 2], Zhenyu Qi[1, 2], Dan Wang[1, 2], and Liping Chen[1, 2,*]

[1]Department of Horticulture, College of Agriculture and Biotechnology,

Zhejiang University, Hangzhou, 310058, P. R. China

[2]Zhejiang Provincial Key Laboratory of Horticultural Plant Integrative Biology,

Zhejiang University, Hangzhou, 310058, P. R. China

[*]Corresponding. chenliping@zju.edu.cn

Supplementary Table S1. All primers used in this paper.

Primers/Adapters	Sequences (5’-3’)	Primers/Adapters	Sequences (5’-3’)
*E*coRI adapter	CTCGTAGACTGCGTACC; AATTGGTACGCAGTCTAC	*Hpa* II/*Msp* I adapter	GATCATGAGTCCTGCT; CGAGCAGGACTCATGA
ECOR0	GACTGCGTACCAATTCA	H/M0	ATCATGAGTCCTGCTCGG
ECOR1	GACTGCGTACCAATTCAAA	H/M1	ATCATGAGTCCTGCTCGGTAA
ECOR2	GACTGCGTACCAATTCAAC	H/M2	ATCATGAGTCCTGCTCGGTAC
ECOR3	GACTGCGTACCAATTCAAG	H/M3	ATCATGAGTCCTGCTCGGTAG
ECOR4	GACTGCGTACCAATTCAAT	H/M4	ATCATGAGTCCTGCTCGGTAT
ECOR5	GACTGCGTACCAATTCACC	H/M5	ATCATGAGTCCTGCTCGGTCC
ECOR6	GACTGCGTACCAATTCACA	H/M6	ATCATGAGTCCTGCTCGGTCA
ECOR7	GACTGCGTACCAATTCACG	H/M7	ATCATGAGTCCTGCTCGGTCG
ECOR8	GACTGCGTACCAATTCACT	H/M8	ATCATGAGTCCTGCTCGGTCT
ECOR9	GACTGCGTACCAATTCAGA	H/M9	ATCATGAGTCCTGCTCGGTGA
ECOR10	GACTGCGTACCAATTCAGC	H/M10	ATCATGAGTCCTGCTCGGTGG
ECOR11	GACTGCGTACCAATTCAGG	H/M11	ATCATGAGTCCTGCTCGGTGC
ECOR12	GACTGCGTACCAATTCAGT	H/M13	ATCATGAGTCCTGCTCGGTTA
ECOR13	GACTGCGTACCAATTCATA	H/M14	ATCATGAGTCCTGCTCGGTTC
ECOR14	GACTGCGTACCAATTCATC	H/M15	ATCATGAGTCCTGCTCGGTTG
ECOR15	GACTGCGTACCAATTCATT	H/M16	ATCATGAGTCCTGCTCGGTTT
ECOR16	GACTGCGTACCAATTCATG		
Primers for genome walking			
SP1-dDMF5	AGAACAGCATACACTAACCCACCA	SP2-dDMF5	CCTTAGGAGTGCCATCCTCCATAT
SP3-dDMF5	TCCTTGTTTGTGGGTTCATTCG	SP1-dDMF7	GAGATTCTTATCGAGGAGTC
SP2-dDMF7	CGAACGTTCAGCCTGTCAAC	SP3-dDMF7	GTTCAGCCTGTCAACAGTCC

SP1-dDMF12	CTCCTTCCGAGATCTGATCAAAAGC	SP2-dDMF12	ACTCATCTTTCATCTCCAGAGCCTTA
SP3-dDMF12	TCTCTCCACTAAATCCACCGCCCTTC	SP1-dDMF30	TGCCTTTCCATGTAGAGGGGACCGAA
SP2-dDMF30	GGGGACCGAACCAAGCCCATCGTCAT	SP3-dDMF30	CGGCCAGATTCCGGTATCGACTATAC
5’SP1-uDMF9	TGTTCGGAAGTAGTGCTGCAATGTAA	5’SP2-uDMF9	GTGAATTGCAGAATCCCGTGAACCAT
5’SP3-uDMF9	CACAAATCGTCGTCCCCCCATCCTCT	3’SP1-uDMF9	CGAGAGGATGGGGGGACGACGATT
3’SP2-uDMF9	ATTCACACCAAGTATCGCATTTCG	3’SP3-uDMF9	GCACTACTTCCGAACAAACACCGT
Primers for qRT-PCR			
25SF	CGGTTCCTCTCGTACTAGGTTGA	25SR	CCGTCGTGAGACAGGTTAGTTTT
dDMF5F	CCGGAATCGAAGGTGTTAAT	dDMF5R	TGTCGGAGTTCAGGGTGTTA
dDMF7F	GACTGTTGACAGGCTGAACG	dDMF7R	CTCAGCGATTGATTCCAAGA
dDMF12F	AGGAAGAAAGCGACTGGTGT	dDMF12R	CAGAGAGAATCTGCGGATCA
dDMF30F	CGGTTAAGCTCCAAACCACT	dDMF30R	ATAACCGTACCGGCTCCATA
uDMF32F	GGAAAGTGCAACGTGAGATG	uDMF32R	GAGGGCACACAACCATTACA
Primers for bisulfite sequencing			
dDMF5F	AATTTTGTTTTTTGATAGTTTTGTAG	dDMF5R	TAAAAATACCATCCTCCATATATTC
dDMF7F	TTAGAAGGTGTTTTTTGTTTTTGATAT	dDMF7R	ACTATTATAACCTCTATCCACAAAATCC
dDMF12F	AGATTAAAGTTATAGATTTTTTTATTAAAT	dDMF12R	AAACAAACACTCCAAAACTAAACTAAC
dDMF30F	GGTTAAGTTTTAAATTATTTTTTTT	dDMF30R	ACCCTTATTAAAAACCCTAACACC
uDMF9F	AGGATTTTGGGTTATTAAGAGTTTTT	uDMF9R	CCAAAACTTCAATTTTAATTAAATC

Supplementary Table S2. Analysis of DNA methylation levels detected by MSAP in TTT and three self-grafted plants TTT+TTT

MSAP Band type	TTT	TTT+TTT1	TTT+TTT2	TTT+TTT3
**I (unmethylation)**	591	588	591	589
**II (CHG methylation)**	188	191	185	192
**III (CG methylation)**	343	344	347	344
**IV (CG/CHG methylation)**	7	6	6	4
**Total Bands**	1129			
**Total methylated bands[a]**	538	541	538	540
**Total methylation ratio (%)[b]**	47.65	47.92	47.65	47.83
**Full methylated bands[c]**	350	350	353	348
**Full methylated ratio (%)[d]**	31.00	31.00	31.27	30.82

[a]Total methylated bands = II + III + IV

[b]Total methylated ratio = [(II + III + IV)/ (I + II + III + IV)] × 100 %

[c]Fully methylated bands = III + IV

[d]Fully methylated ratio = [(III + IV)/ (I + II + III + IV)] × 100

Supplementary Table S3. Analysis of cytosine methylation pattern variations of three self-grafted plants TTT+TTT

Pattern	Class	Banding pattern				TTT+TTT		
		TTT		TTT+TTT				
		*Hpa*II	*Msp*I	*Hpa*II	*Msp*I	TTT+TTT1	TTT+TTT 2	TTT+TTT 3
**No change**	A	1	1	1	1	587	585	588
	B	1	0	1	0	186	183	186
	C	0	1	0	1	342	341	342
	D	0	0	0	0	5	3	1
	Total I					1120	1112	1117
						99.20%	98.49%	98.94%
**Methylation**	E	1	1	1	0	3	0	2
	F	1	1	0	1	2	5	0
	G	1	1	0	0	0	1	1
	H	1	0	0	0	1	2	1
	I	0	1	0	0	0	0	1
	Total II					6	8	5
						0.53%	0.71%	0.44%
**Demethylation**	J	1	0	1	1	0	3	1
	K	0	1	1	1	1	2	0
	L	0	0	1	1	0	1	0
	M	0	0	1	0	2	2	4
	N	0	0	0	1	0	1	2
	Total III					3	9	7
						0.27%	0.80%	0.62%
	Total variation in pattern (Total II+Total III)					0.80%	1.51%	1.06%

A score of 1 and 0 represents presence and absence of bands, respectively. Values in parentheses indicate percentage of bands in each pattern which was determined by dividing number of bands in each pattern by total number of bands in all three patterns.

Supplementary Table S4. BLAST results of differentially DNA methylated fragments and their location on genome.

MSAP fragments	Methylation status after grafting	Sequence Size (bp)	Gene	Locus ID Brassica rapa (Arabidopsis thaliana)	E value	Position
uniform DMFs						
uDMF2	Demethylated	103	Transposon	-	2e-96	-
uDMF3	Demethylated	125	Transposon	-	e-89	-
uDMF4	Demethylated	428	Transposon	-	e-139	-
uDMF6	Methylated	210	Duplicated homeodomain-like superfamily protein, myb family transcription factor	Bra034132 (At3g10595)	e-113	exon
uDMF7	Methylated	485	Transposon	-	0.0	-
uDMF9	Methylated	370	Transposon	-	0.0	-
uDMF10	Methylated	65	Protein of unknown function	Bra001185 (At3g06150)	2e-29	intron
uDMF12	Methylated	151	Leucine-rich repeat protein kinase family protein	Bra005371 (At2g35040)	1e-20	intron
uDMF14	Demethylated	318	Transposon	-	e-142	-
uDMF15	Demethylated	209	Tetratricopeptide repeat (TPR)-like superfamily protein	Bra038468 (At4g16470)	6e-63	exon
uDMF16	Demethylated	104	Member of Putative potassium proton antiporter family	Bra034496 (At5g51710)	5e-37	exon
uDMF18	Demethylated	207	Protein of unknown function	Bra009206 (At5g06560)	3e-18	exon
uDMF19	Methylated	337	Encodes a protein with similarity to formins that is involved in cytokinesis	Bra002969	2e-85	exon

				(At5g54650)		
uDMF20	Demethylated	149	Transposon	-	5e-07	-
uDMF21	Demethylated	165	Transposon	-	2e-86	-
uDMF22	Demethylated	149	Transposon	-	e-124	-
uDMF23	Demethylated	238	Protein of unknown function	Bra033264 (At1g01440)	5e-13	exon
uDMF25	Demethylated	100	Jasmonate-zim-domain protein 1	Bra016520 (At1g19180)	1e-19	exon
uDMF26	Methylated	278	Protein of unknown function	Bra021211 (At3g16900)	2e-78	exon
uDMF27	Demethylated	263	Encodes a protein that appears to have 1-amino-cyclopropane-1-carboxylic acid oxidase activity based on mutant analyses.	Bra019734 (At1g12010)	2e-23	exon
uDMF28	Demethylated	208	No similarity	-	-	-
uDMF29	Demethylated	186	No similarity	-	-	-
uDMF30	Demethylated	156	Protein of unknown function	Bra022787 (At2g30270)	e-69	intron
uDMF31	Methylated	364	ARIADNE 9 (ARI9), zinc ion binding	Bra022865 (At2g31770)	e-104	exon
uDMF32	Demethylated	139	Gibberellin-regulated family protein	Bra019917 (At2g39540)	2e-49	exon
distinctive DMFs						
dDMF2	Demethylated	93	No similarity	-	-	-
dDMF3	Demethylated	72	No similarity	-	-	-
dDMF5	Demethylated	242	Zinc knuckle (CCHC-type) family protein	Bra002717 (At2g07760)	e-132	exon
dDMF6	Demethylated	232	Similar to myrosinase binding proteins which may be involved in	Bra018942	5e-73	intron

			metabolizing glucosinolates and forming defense compounds to protect against herbivory. Also similar to lectins and other agglutinating factors	(At1g52030)		
dDMF7	Methylated	225	Encodes catalytic subunit of serine/threonine protein phosphatase 2A	Bra001770 (At3g19980)	9e-50	exon
dDMF8	Methylated	183	Hydroxyproline-rich glycoprotein family protein	Bra011966 (At2g28240)	4e-85	intron
dDMF9	Methylated	95	Transposon	-	3e-47	-
dDMF10	Demethylated	254	No similarity	-	-	-
dDMF11	Methylated	193	RAB GDP DISSOCIATION INHIBITOR 2	Bra014523 ()	e-105	exon
dDMF12	Demethylated	340	Encodes the putative delta subunit of the AP(adaptor protein)-3 complex and plays a role in vacuolar function	Bra022309 (At1g48760)	0.0	exon
dDMF14	Methylated	106	No similarity	-	-	-
dDMF15	Methylated	77	RNA-binding (RRM/RBD/RNP motifs) family protein	Bra021645 (At5g03495)	1e-36	exon
dDMF16	Methylated	86	Protein of unknown function	Scaffold000424	2e-20	-
dDMF17	Demethylated	72	Encodes a homolog of animal DJ-1 superfamily protein	Bra001577 (At3g14990)	9e-08	exton
dDMF18	Methylated	72	RING/U-box protein; FUNCTIONS IN: DNA binding	Bra001159 (At3g05670)	9e-07	exton
dDMF19	Methylated	73	Member of MEKK subfamily	Bra026395 (At4g26890)	6e-14	exon
dDMF20	Methylated	116	DNA binding with one finger 4.7 (DOF4.7)	Bra011800 (At4g38000)	7e-49	exon
dDMF21	Methylated	465	Phospholipase-like protein (PEARLI 4) family	Bra007128	0.00	extron

				(At2g20960)		
dDMF22	Demethylated	292	Plant protein of function unknown	Bra031558 (At1g07220)	e-164	exon
dDMF23	Methylated	247	Disease resistance protein (TIR-NBS-LRR class), putative	Bra036413 (At5g11250)	6e-85	exon
dDMF24	Demethylated	277	Do similarity	-	-	-
dDMF27		147	Unknown protein	Bra018031 (At3g48860)	4e-20	exon
dDMF30	Demethylated	93	Subtilisin-like serine endopeptidase family protein	Bra026561 (At5g67090)	5e-46	exon
dDMF32	Demethylated	201	No similarity	-	-	-
dDMF33	Demethylated	154	Transposon	-	6e-50	-
dDMF34	Demethylated	136	No similarity	-	-	-
dDMF37	Demethylated	247	Transposon	-	6e-85	-
dDMF39	Methylated	296	Transposon	-	7e-42	-
dDMF40	Methylated	278	Protein of unknown function	Bra036003 (At5g43950)	2e-76	exon

Supplementary Table S5. List of differentially expressed siRNAs between TTT and GS1, and their matched DMFs.

siRNA		DMF	identity	Alignment length	DMF start	DMF end	e-values
siR1	CCATCGAGTCTTTGAACGCAAGTTG	uDMF9	100	25	186	162	2.00E-10
siR2	TGAACCATCGAGTCTTTGAACGCAA	uDMF9	100	25	190	166	2.00E-10
siR3	ACCATCGAGTCTTTGAACGCAAGTT	uDMF9	100	25	187	163	2.00E-10
siR4	AATTGCAGAATCCCGTGAACCATCG	uDMF9	100	25	205	181	2.00E-10
siR5	CGATGAAGAACGTAGCGAAATGCG	uDMF9	100	24	218	241	6.00E-10
siR6	CGATACTTGGTGTGAATTGCAGAA	uDMF9	100	24	196	219	6.00E-10
siR7	ACTTGGTGTGAATTGCAGAATCCC	uDMF9	100	24	192	215	6.00E-10
siR8	TTGGTGTGAATTGCAGAATCCCGT	uDMF9	100	24	190	213	6.00E-10
siR9	ATTGCAGAATCCCGTGAACCATCG	uDMF9	100	24	181	204	6.00E-10
siR10	TTGCAGAATCCCGTGAACCATCGA	uDMF9	100	24	180	203	6.00E-10
siR11	TGAACCATCGAGTCTTTGAACGCA	uDMF9	100	24	167	190	6.00E-10
siR12	AACCATCGAGTCTTTGAACGCAAG	uDMF9	100	24	165	188	6.00E-10
siR13	ACCATCGAGTCTTTGAACGCAAGT	uDMF9	100	24	164	187	6.00E-10
siR14	CCATCGAGTCTTTGAACGCAAGTT	uDMF9	100	24	163	186	6.00E-10
siR15	CATCGAGTCTTTGAACGCAAGTTG	uDMF9	100	24	162	185	6.00E-10
siR16	ATCGAGTCTTTGAACGCAAGTTGC	uDMF9	100	24	161	184	6.00E-10
siR17	CCTTCTGGCCGAGGGCACGTCTGC	uDMF9	100	24	129	152	6.00E-10
siR18	CTCGAGAGGATGGGGGACGACGA	uDMF9	100	24	91	114	6.00E-10
siR19	CCTTAGCTCGGATTTTGGCCAACC	uDMF9	100	24	26	49	6.00E-10
siR20	CCCTTAGCTCGGATTTTGGCCAAC	uDMF9	100	24	25	48	6.00E-10
siR21	CGCCCTTAGCTCGGATTTTGGCCA	uDMF9	100	24	26	43	6.00E-10
siR22	ATCGAGTCTTTGAACGCAAGTTG	uDMF9	100	23	184	162	2.00E-09
siR23	GTGTGAATTGCAGAATCCCGTGA	uDMF9	100	23	210	188	2.00E-09
siR24	CCTTCTGGCCGAGGGCACGTCTG	uDMF9	100	23	152	130	2.00E-09
siR25	ACCATCGAGTCTTTGAACGCAAG	uDMF9	100	23	187	165	2.00E-09
siR26	TCGAGTCTTTGAACGCAAGTTGC	uDMF9	100	23	183	161	2.00E-09
siR27	CGGATATCTCGGCTCTCGCATCG	uDMF9	100	23	262	240	2.00E-09
siR28	GCCCTTAGCTCGGATTTTGGCCA	uDMF9	100	23	24	46	2.00E-09
siR29	TGAACCATCGAGTCTTTGAACGC	uDMF9	100	23	190	168	2.00E-09
siR30	GCTCTCGCATCGATGAAGAACGT	uDMF9	100	23	251	229	2.00E-09
siR31	CCATCGAGTCTTTGAACGCAAGT	uDMF9	100	23	186	164	2.00E-09
siR32	CGATGAAGAACGTAGCGAAATG	uDMF9	100	22	241	220	8.00E-09
siR33	GTGTGAATTGCAGAATCCCGTG	uDMF9	100	22	210	189	8.00E-09
siR34	TCGAGTCTTTGAACGCAAGTTG	uDMF9	100	22	183	162	8.00E-09
siR35	GCCCTTAGCTCGGATTTTGGCC	uDMF9	100	22	24	45	8.00E-09
siR36	CGAGTCTTTGAACGCAAGTTGC	uDMF9	100	22	182	161	8.00E-09
siR37	ACTTGGTGTGAATTGCAGAATC	uDMF9	100	22	215	194	8.00E-09
siR38	GAGTCTTTGAACGCAAGTTGCG	uDMF9	100	22	181	160	8.00E-09
siR39	CCAAAATCCGAGCTAAGGGCGC	uDMF9	100	22	43	22	8.00E-09

siR40	ATTGCAGAATCCCGTGAACCAT	uDMF9	100	22	204	183	8.00E-09
siR41	ATCTCGGCTCTCGCATCGATGA	uDMF9	100	22	257	236	8.00E-09
siR42	CCATCGAGTCTTTGAACGCAAG	uDMF9	100	22	186	165	8.00E-09
siR43	GCAACGGATATCTCGGCTCTCG	uDMF9	100	22	266	245	8.00E-09
siR44	CGATACTTGGTGTGAATTGCAG	uDMF9	100	22	219	198	8.00E-09
siR45	CGGATATCTCGGCTCTCGCATC	uDMF9	100	22	262	241	8.00E-09
siR46	AGTCTTTGAACGCAAGTTGCGC	uDMF9	100	22	180	159	8.00E-09
siR47	CAGAAGGCTTGGGGCGCAACTT	uDMF9	100	22	146	167	8.00E-09
siR48	CATCGAGTCTTTGAACGCAAGT	uDMF9	100	22	185	164	8.00E-09
siR49	AGAAGGCTTGGGGCGCAACTTG	uDMF9	100	22	147	168	8.00E-09
siR50	CACGTCTGCCTGGGTGTCACA	uDMF9	100	21	137	117	3.00E-08
siR51	GCATCGATGAAGAACGTAGCG	uDMF9	100	21	245	225	3.00E-08
siR52	GTGTGAATTGCAGAATCCCGT	uDMF9	100	21	210	190	3.00E-08
siR53	CGAGTCTTTGAACGCAAGTTG	uDMF9	100	21	182	162	3.00E-08
siR54	GATGAAGAACGTAGCGAAATG	uDMF9	100	21	240	220	3.00E-08
siR55	AGCGAAATGCGATACTTGGTG	uDMF9	100	21	228	208	3.00E-08
siR56	CTCGCATCGATGAAGAACGTA	uDMF9	100	21	248	228	3.00E-08
siR57	AATTGCAGAATCCCGTGAACC	uDMF9	100	21	205	185	3.00E-08
siR58	GGATATCTCGGCTCTCGCATC	uDMF9	100	21	261	241	3.00E-08
siR59	TGCGTTCAAAGACTCGATGGT	uDMF9	100	21	167	187	3.00E-08
siR60	TCGGCTCTCGCATCGATGAAG	uDMF9	100	21	254	234	3.00E-08
siR61	GCACGTCTGCCTGGGTGTCAC	uDMF9	100	21	138	118	3.00E-08
siR62	ATACTTGGTGTGAATTGCAGA	uDMF9	100	21	217	197	3.00E-08
siR63	CATCGAGTCTTTGAACGCAAG	uDMF9	100	21	185	165	3.00E-08
siR64	GGGGGGACGACGATTTGTGAC	uDMF9	100	21	102	122	3.00E-08
siR65	ATCTCGGCTCTCGCATCGATG	uDMF9	100	21	257	237	3.00E-08
siR66	TCGAGTCTTTGAACGCAAGTT	uDMF9	100	21	183	163	3.00E-08
siR67	ATCGAGTCTTTGAACGCAAGT	uDMF9	100	21	184	164	3.00E-08
siR68	GAGTCTTTGAACGCAAGTTGC	uDMF9	100	21	181	161	3.00E-08
siR69	GCGTTCAAAGACTCGATGGTT	uDMF9	100	21	168	188	3.00E-08
siR70	AGTCTTTGAACGCAAGTTGCG	uDMF9	100	21	180	160	3.00E-08
siR71	TGAACCATCGAGTCTTTGAAC	uDMF9	100	21	190	170	3.00E-08
siR72	TCTCGGCTCTCGCATCGATGA	uDMF9	100	21	256	236	3.00E-08
siR73	CCTTAGCTCGGATTTTGGCCA	uDMF9	100	21	26	46	3.00E-08
siR74	CCCGTGAACCATCGAGTCTTT	uDMF9	100	21	194	174	3.00E-08
siR75	TGTGAATTGCAGAATCCCGT	uDMF9	100	20	209	190	1.00E-07
siR76	AGTCTTTGAACGCAAGTTGC	uDMF9	100	20	180	161	1.00E-07
siR78	TGAAGAACGTAGCGAAATGC	uDMF9	100	20	238	219	1.00E-07
siR79	AACGACTCTCGGCAACGGAA	uDMF9	100	19	277	259	4.00E-07
siR80	TAAAACGACTCTCGGCAACT	uDMF9	100	19	280	262	4.00E-07
siR81	GTGATGTATGCAGCTGAGGCA	dDMF16	100	21	28	8	3.00E-08
siR82	ATTGTGGAGAGAAAGTGAGAGATA	dDMF30	100	17	128	112	9.00E-06

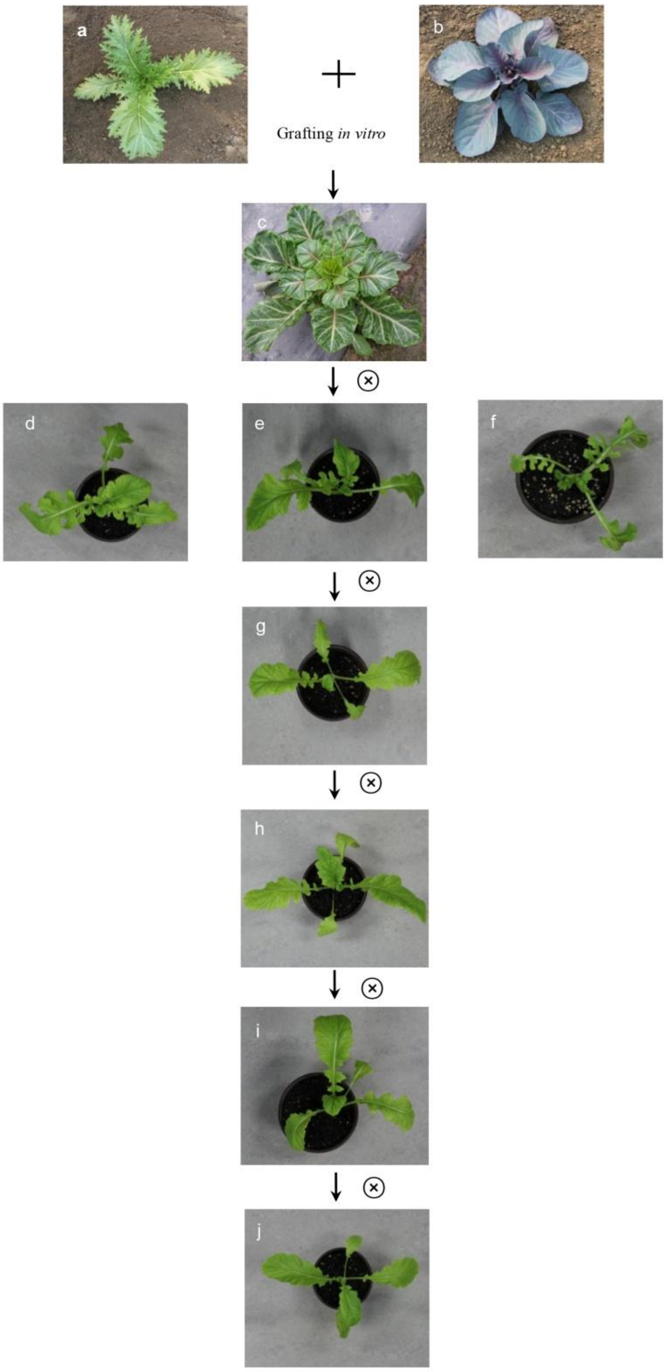

Supplementary Figure S1. Diagram of grafting and creating the sexual chimera progenies
(a) TTT; (b) CCC; (c) TTC;(d) GS1; (e) GS1; (f) GS1; (g) GS2; (h) GS3; (i) GS4; (j) GS5

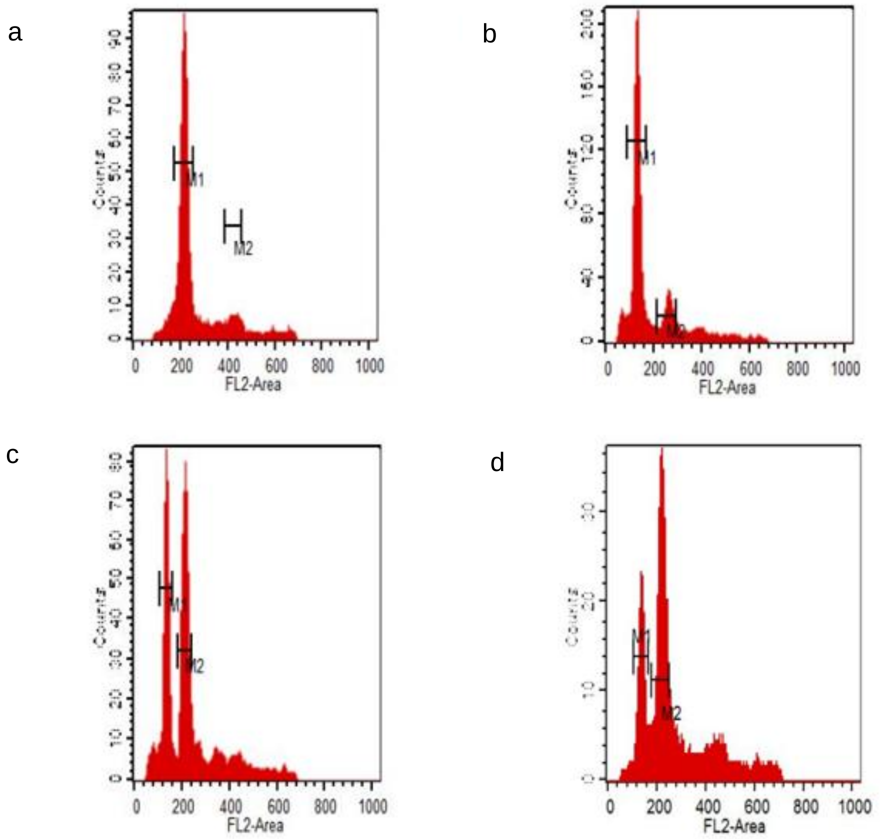

Supplementary Figure S2. Flow cytometry analysis. (a) DNA content of TTT; (b) DNA content of CCC; (c) DNA content of TTC; (d) DNA content of the mechanical mixture of TTT and CCC.

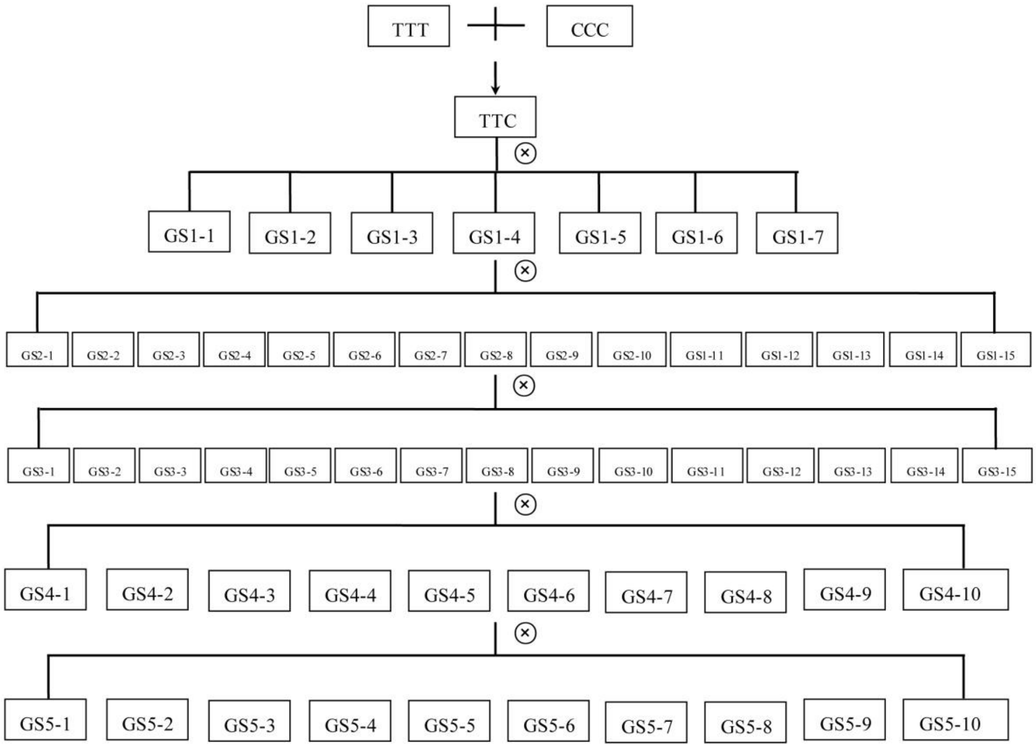

Supplementary Figure S3. The lineage of all plants used for MSAP analysis.
